# Genome mining for anti-CRISPR operons using machine learning

**DOI:** 10.1093/bioinformatics/btad309

**Published:** 2023-05-09

**Authors:** Bowen Yang, Minal Khatri, Jinfang Zheng, Jitender Deogun, Yanbin Yin

**Affiliations:** Department of Food Science and Technology, Nebraska Food for Health Center, University of Nebraska—Lincoln, Lincoln, NE 68508, United States; School of Computing, University of Nebraska, Lincoln, NE 68588, United States; Department of Food Science and Technology, Nebraska Food for Health Center, University of Nebraska—Lincoln, Lincoln, NE 68508, United States; School of Computing, University of Nebraska, Lincoln, NE 68588, United States; Department of Food Science and Technology, Nebraska Food for Health Center, University of Nebraska—Lincoln, Lincoln, NE 68508, United States

## Abstract

**Motivation:**

Encoded by (pro-)viruses, anti-CRISPR (Acr) proteins inhibit the CRISPR-Cas immune system of their prokaryotic hosts. As a result, Acr proteins can be employed to develop more controllable CRISPR-Cas genome editing tools. Recent studies revealed that known *acr* genes often coexist with other *acr* genes and with phage structural genes within the same operon. For example, we found that 47 of 98 known *acr* genes (or their homologs) co-exist in the same operons. None of the current Acr prediction tools have considered this important genomic context feature. We have developed a new software tool **AOminer** to facilitate the improved discovery of new Acrs by fully exploiting the genomic context of known *acr* genes and their homologs.

**Results:**

AOminer is the first machine learning based tool focused on the discovery of Acr operons (AOs). A two-state HMM (hidden Markov model) was trained to learn the conserved genomic context of operons that contain known *acr* genes or their homologs, and the learnt features could distinguish AOs and non-AOs. AOminer allows automated mining for potential AOs from query genomes or operons. AOminer outperformed all existing Acr prediction tools with an accuracy = 0.85. AOminer will facilitate the discovery of novel anti-CRISPR operons.

**Availability and implementation:**

The webserver is available at: http://aca.unl.edu/AOminer/AOminer_APP/. The python program is at: https://github.com/boweny920/AOminer.

## 1 Introduction

Anti-CRISPR (Acr) proteins have attracted a great attention for its application in genome editing (Bondy-Denomy et al. 2013; Nakamura et al. 2019). A total of 98 Acr proteins have been experimentally characterized. Notably, most Acrs are orphan genes (Yin and Fischer 2008), as no significant sequence similarity was found between the 98 Acrs. In addition, the 98 known Acrs were shown to inhibit only 11/33 CRISPR-Cas subtypes suggesting that the experimentally characterized Acrs only represent a tiny tip of an iceberg of the possible anti-CRISPR diversity in nature.

Six bioinformatics tools are available for automated Acr discovery: AcRanker (Eitzinger et al. 2020), AcrFinder (Yi et al. 2020), PaCRISPR (Wang et al. 2020), DeepAcr (Wandera et al. 2022), AcrNET (Li et al. 2022), and AcrPred (Dao et al. 2023). There is one important genomic context feature, however, that has never been employed in these tools: the co-localization of *acr* genes with other genes. For example, 42 of the 98 known *acr* genes reside in short gene operons containing 32 multiple types of *acr* genes (Supplementary Table S1). Also, 41 of the 98 *acr* genes have putative *aca* (*acr-*associated HTH domain-containing protein) genes nearby (Yin et al. 2019). Additionally, *acr* genes can also co-localize with conserved phage genes [e.g. capsid, terminase, lysozyme, tail, helicase (León et al. 2021)] and functionally unknown genes in the gene neighborhood. Therefore, the genomic context of *acr* genes could be fully exploited in a machine learning model for improved discovery of Acrs.

Here, we present AOminer focusing on the discovery of Acr operons by learning the conserved genomic context of *acr* genes. The predicted Acr operons have a higher chance to contain putative Acrs than other regions in the query genome, and could be further analyzed by other bioinformatics tools or by experimental approaches for new Acrs.

## 2 Algorithm

**Figure 1.**
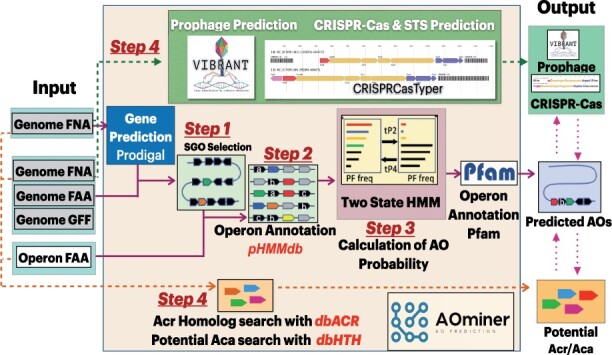
AOminer algorithm. Refer to Supplementary Methods for step specifics.

AOminer accepts FASTA sequences of whole genomes/contigs as well as individual gene clusters/operons as input. The sequences will be processed with the following steps (Fig. 1):

Step 1: Prodigal (Hyatt et al. 2010) predicts genes, and short-gene operons (SGOs) are defined (see Supplementary Method). Users can also input their own SGOs or non-operon gene clusters.

Step 2: The SGOs will be annotated with a protein family profile HMM (pHMM) database using hmmscan (Finn et al. 2011). This pHMMdb contains 2030 pHMMs of AO protein families (AOPFs) and 1218 non-AOPFs that were built based on dbAO (see Supplementary Method and Table S2). The dbAO consists of 12 582 nonredundant Acr operons (AOs) that must contain homologs of 98 known Acr proteins collected from six phage and prophage genome databases.

Step 3: Each annotated SGO will go through our two-state HMM to receive a prediction score. SGOs that have a prediction score >3 will be returned to the users as AOs (see Supplementary Method). The key idea of the two-state HMM is that AOs of dbAO have different protein family profiles (e.g. more AOPFs and less non-AOPFs) compared to non-AOs, and that such difference can be modeled for new AO discovery.

Step 4: AOminer will also scan the query genome for Acr and Aca homologs using the built-in dbACR and dbHTH (Supplementary Method). Users also have the option to identify prophages, CRISPR-Cas systems, and self-targeting spacers (STSs) in their query genomes.

## 3 Implementation

*Standalone program*: AOminer was written in Python. For contig/genome input, a FNA file is expected, and annotation files (FAA, GFF) are optional. For operon or gene cluster input, AOminer expects a FAA file with proteins following their order in the DNA sequence. Users can provide their own known Acr sequence and HTH domain databases.

The output of AOminer includes: (i) table of all predicted AOs (example in Supplementary Table S3-1); (ii) table of all predicted CRISPR-Cas systems (Supplementary Table S3-2); and (iii) table of predicted prophage regions (Supplementary Table S3-3).

*Web server*: A web server was developed for users without programing experience. The server was constructed using the Django framework.

## 4 Performance evaluation

To evaluate the performance of AOminer, we split the 12 582 AOs in dbAO (Supplementary Table S2) into dbAO-Train and dbAO-Test. Specifically, a total of 10 481 AOs in dbAO-Train contain homologs of 77 known Acrs published before the year 2020; 2101 AOs in dbAO-Test (Supplementary Table S4) contain homologs of 21 known Acrs published in 2021 and 2022 (Supplementary Table S5). Note that dbAO-Train and dbAO-Test can still share protein families but the Acr homologs of the two datasets are identified based on homology to two sets of published Acrs (i.e. 77 versus 21). After retraining the two-state HMM using dbAO-Train and testing it on dbAO-Test, AOminer was able to find 1791 out of the 2101 AOs with prediction score >3 (recall is 0.852). The dbAO-Test data were also run on AcrFinder, AcRanker, AcrPred, and PaCRISPR, which all had a much lower recall than AOminer (Supplementary Table S6). Unlike AOminer, all these tools are designed to directly predict Acr proteins instead of their operons. Therefore, the predicted Acr proteins from these tools were located in SGOs to be considered as AOs (true positives). To account for the difference in the pipeline design and output/input format, each tool was run with an individualized evaluation process (see Supplementary Method). An additional test was conducted on 10 unpublished but experimentally characterized Acrs kindly provided by Dr. Karen Maxwell, showing a recall = 90% (i.e. 9 was found by AOminer). Due to the lack of true negative data, a precision could not be calculated as what has been published for other tools (Eitzinger et al. 2020; Wang et al. 2020).

## Supplementary Material

btad309_Supplementary_Data
