# Supplementary material for: Genome mining for anti-CRISPR operons using machine learning
Source: Bioinformatics. 2023 May 9;39(5):btad309. doi: 10.1093/bioinformatics/btad309 (PMC10196667; doi:10.1093/bioinformatics/btad309)
Supplement: btad309_Supplementary_Data [file btad309_supplementary_data.zip › Supplemental Materials and Methods.docx]

# Supplemental Methods

**Genome mining for anti-CRISPR operons using machine learning**

*Bowen Yang1,*, Minal Khatri2,*, Jinfang Zheng1, Jitender Deogun2, and Yanbin Yin1,$*

*1Nebraska Food for Health Center, Department of Food Science and Technology, University of Nebraska - Lincoln, Lincoln, NE, 68508, USA*

*2School of Computing, University of Nebraska, Lincoln, NE 68588, USA*

# To whom correspondence should be addressed

** co-first authors*

Yanbin Yin

Tel: 1-402-472-4303

Email: [yyin@unl.edu](mailto:yyin@unl.edu)

**1. Data collection**

To identify Acr homologs and operons, we used the following viral and prokaryotic genomic data:

1. 21,852 complete bacterial genomes, 1,289 archaeal genomes, and 11,684 viral genomes of the RefSeq database (O'Leary, et al., 2016).
2. 4,644 species representative metagenome assembled genomes (MAGs) of the Unified Human Gastrointestinal Genome (UHGG) database (Almeida, et al., 2021).
3. 189,680 viral contigs of the Metagenomic Gut Virus (MGV) database (Nayfach, et al., 2021).
4. 2,377,994 viral contigs of the IMGVR database (Roux, et al., 2021).
5. 17,694 phage contigs of the INfrastructure for a PHAge REference Database (INPHARED) (Cook, et al., 2021).
6. 142,809 contigs of the Gut Phage Database (GPD) (Unterer, et al., 2021).

**2. Building dbAO and AOPF profile HMMs (positive training data)**

The prokaryotic genomes were searched for prophage regions by running VIBRANT (Kieft, et al., 2020). The predicted prophage regions along with the collected viral genomes/contigs was processed with the following steps:

1. Anti-CRISPR Short Gene Operons (SGOs) were scanned using 4 criteria: (i) At least one Acr homologous gene found in the SGO using blastp [E-value < 1e-3 and coverage > 80%]; (ii) All genes on the same strand; (iii) All intergenic distances < 250bp; and (iv) All genes have protein sequence length < 200aa (except that when the Acr homologs are homologous to known Acrs that are longer than 200aa, e.g., AcrIIIB1 [249aa], AcrVA2 [322aa]).
2. To remove duplicates, we used Usearch (Edgar, 2010) and clustered all Acr SGOs that had identity > 95% and target coverage > 90%. This resulted in 12,582 non-redundant Acr operons (AOs), forming the dbAO (**Table S2**).
3. To gather conserved protein families in dbAO, proteins of all SGOs were clustered using Usearch with identity > 40% and target coverage > 60%. The resulting protein clusters with >= 5 proteins were considered to be conserved protein families (AOPFs). In total 2,032 AOPFs remained.
4. A profile HMM (pHMM) was built for each AOPF. All protein sequences of each AOPF were aligned using MUSCLE (Edgar, 2004); hmmbuild (Finn, et al., 2011) was then used to construct the pHMMs. This resulted in a total of 2,032 AOPF pHMMs.

**3. Building of dbNonAO and non-AOPF pHMMs (negative training data)**

A collection of non-Acr operons (non-AOs) and associated protein families (non-AOPFs) were also constructed for the training of AOminer. To have a more balanced AO and non-AO data sizes, we randomly selected the same number of (pro)phage genomes as those that contain the 12,582 AOs of dbAO. The selected genomes were then processed with the following steps:

1. The non-Acr SGOs were selected meeting all 5 criteria: (i) No Acr homologous genes were present in SGOs using blastp search against the 98 known Acrs [E-value < 1e-1 and coverage > 60%]; (ii) No genes matched the AOPFs (see above), determined by running hmmscan with the 2,032 AOPF pHMMs [E-value < 1e-1 and coverage > 40%]; (iii) All genes on the same strand; (iv) All intergenic distances < 250bp; (v) All genes have protein sequence length < 200aa.
2. The non-Acr SGOs was then dereplicated using Usearch [identity > 95% and target coverage > 90%]. This resulted in a total of 6,335 non-redundant non-Acr SGOs (non-AOs), forming the dbNonAO.
3. To collect proteins that prefer not seen in AOs, all proteins of the selected (pro)phage genomes were searched against the 98 known Acrs using blastp [E-value < 1e-1 and coverage > 60%] and against the 2,032 AOPF pHMMs using hmmscan [E-value < 1e-1 and coverage > 40%]. Proteins that did not meet the thresholds were kept as non-AO proteins. We used proteins of the entire genomes instead of those of non-AOs to select non-AO proteins, because non-AOPFs will be used to annotate as many non-conserved proteins in AOs as possible and thus do not have to be restricted in dbNonAO.
4. To generate non-AO protein families, all non-AO proteins from the previous step were clustered using Usearch [identity > 40% and target coverage > 60%]. Clusters >= 5 proteins were considered to be non-AO protein families (non-AOPFs).
5. A pHMM was built for each non-AOPF. All protein sequences of each non-AOPF were aligned using MUSCLE (Edgar, 2004); hmmbuild (Finn, et al., 2011) was then used to construct the pHMMs. This resulted in a total of 10,442 non-AOPF pHMMs.

**4. Construction of the two-state HMM for AO prediction and AO threshold (ThAO) determination**

*Two-state HMM design:*

The training dataset include: (i) AOPFs represent 2,032 protein families (PFs) conserved in AOs; (ii) non-AOPFs represent 10,442 PFs that are not conserved in AOs; (iii) 12,582 AOs of dbAO represent positive training data that contains all the occurrence and abundance information of AOPFs and non-AOPFs, and the co-occurrence information of these PFs in the AOs; and (iv) 6,335 non-AOs of dbNonAOs represent negative data. We split the positive set (12,582 AOs) into a training set (80%) and a validation set (20%). The training set was used to train the HMM model and the validation set was used along with the negative set (6,335 non-AOs) to determine a threshold (ThAO) for the separation of AOs and non-AOs.

***
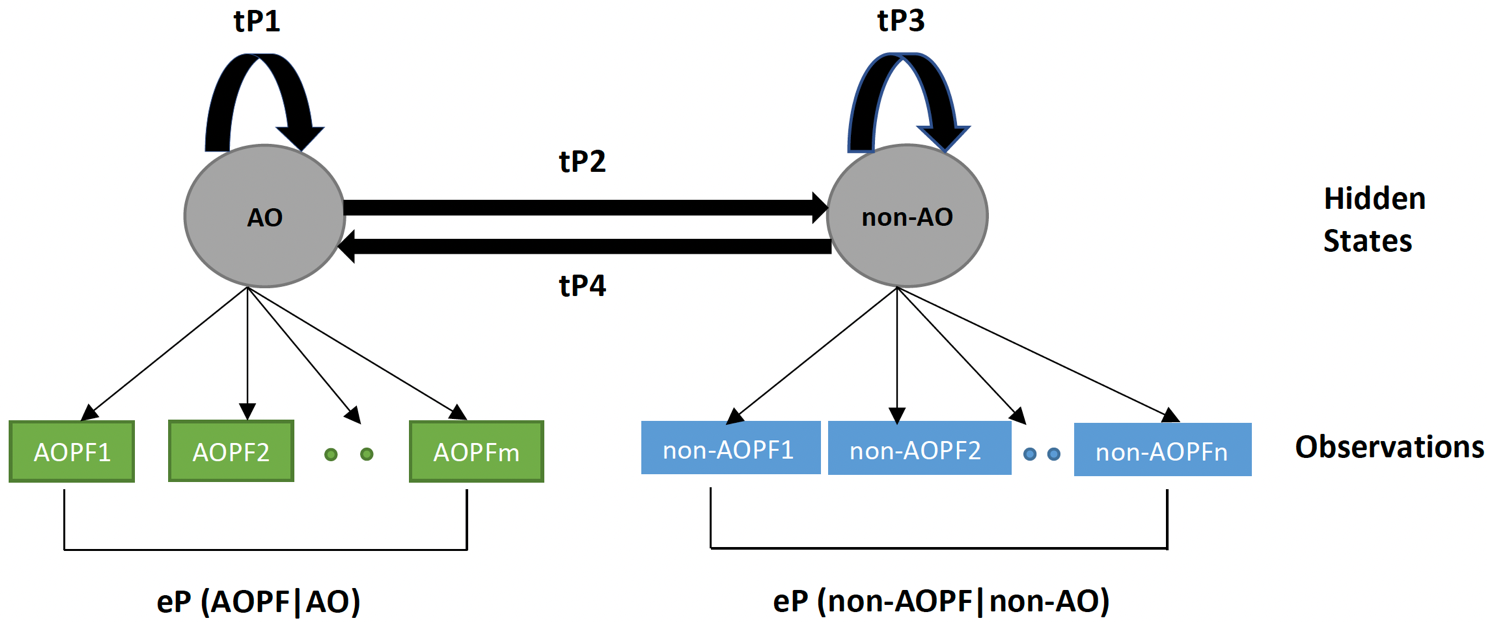
***

***Figure S1.*** *Two-state HMM probability matrix.*

*AOPF represents proteins labeled with AO protein families; non-AOPF represents proteins labeled with non-AO protein families; tP is transition probability between labels; eP(AOPF|AO) is emission probability of AOPFs; eP(non-AOPF|non-AO) is emission probability of non-AOPFs; Hidden States are states of proteins that are not observed directly from labels; Observations are observable labels of proteins within an AO sequence.*

We built a two-state HMM (**Figure S1**) to capture the abundance of AOPFs and non-AOPFs, as well as their co-occurrence in the training AOs. The HMM includes: (i) an AO state represented by the PFs conserved in AOs (ii) a non-AO state represented by the PFs not conserved in AOs. In addition to the two hidden states, we have a set of observations for each state: the set of ‘m’ AOPFs [AOPF1,AOPF2,….,AOPFm] forms the set of observations for the AO state, and the set of ‘n’ non-AOPFs [non-AOPF1,non-AOPF2,…,non-AOPFn ] forms the set of observations for non-AO state.

AOs of the training set were searched against both the AOPF and the non-AOPF pHMM collections using hmmscan (E-value < 1e-3). This annotation process transformed the training AOs into sequences of PF labels. Specifically, to determine the corresponding sequences of (AO or non-AO) states, for proteins in each AO, we assigned AO states to proteins annotated with AOPF pHMMs, and assigned non-AO states to proteins annotated with non-AOPF pHMMs. Proteins without annotations were given an unknown label (NoAO). Since unknowns were not of conserved PFs, they were assigned with a non-AO state. All PF pHMMs present in the annotation of training AOs were collected as a new HMM database, dbPF, which contains a subset of pHMMs from both AOPF and non-AOPF. dbPF will be further used for the annotation of users’ query operons.

*Two-state HMM training (****Figure S1****):*

During the training process, the following three parameters of HMM model were calculated by exanimating all the PF labels assigned in the previous step for all training AOs:

1. Initial probability matrix (): The frequency of an initial state in all training AOs is defined as the initial probability of the state.
2. Transition probability matrix (tP): The co-occurrence among different AOPFs (tP1), among different non-AOPFs (tP3), and between AOPFs and non-AOPFs (tP2 and tP4) were captured by calculating the frequencies of all neighboring PF pairs in all training AOs. The PF pair frequencies are called transition probabilities (tP) in the HMM.
3. Emission probability matrix (eP): The occurrence and abundance of all AOPFs were calculated as the PF frequencies in all the training AOs. Similarly, we calculated the PF frequencies for non-AOPFs. The PF frequencies are called emission probabilities (eP).

*AO probability calculation:*

Once the two state HMM is trained, it could be used to predict the probability of an operon being a novel AO. Given a short gene operon (SGO) we can look up the initial probabilities, emission probabilities and transition probabilities in the two-state HMM, and an overall probability can be calculated.

Given a sequence of proteins, and sequence of states, , the AO probability of the sequence of PF labels can be calculate as follows:

represents initial probability of state,

tP represents transition probability of to

eP( represents emission probability of protein family given state

*AO threshold (ThAO) determination and two-state HMM prediction evaluation:*

For each predicted AO, AOminer will output a probability. Our major goal is to be able to predict high confident novel anti-CRISPR operons, so that experimental experts could further validate the AOs in the wet lab. Therefore, we selected a probability threshold (ThAO) prioritizing on the maximization of sensitivity (recall) and specificity (**Figure S2**). To find the optimal ThAO, we utilized the positive validation set (20% of AOs) and negative validation set (6,335 non-AOs) to determine the probability threshold for the best separation of AOs and non-AOs in the two validation sets. We first calculated the probability of AO/non-AOs in both positive and negative validation set. Maximizing sensitivity and specificity, we found that when using -log(Prob) >3, a best validation accuracy of 86% was obtained (**Table S7**). Therefore the -log(ThAO) was determined to be 3.

***Table S7.*** *Two-State HMM validation results*

| -log(Prob) | Sensitivity (%) | Specificity (%) | Accuracy (%) |
| --- | --- | --- | --- |
| 4 | 89.58 | 81.75 | 84.13 |
| **3** | **97.71** | **80.94** | **86.05** |
| 2 | 97.95 | 79.28 | 84.97 |
| 1 | 98.88 | 60.25 | 72.02 |

AO

Non-AO

**Probability**

Best Threshold

**SGOs**

***Figure S2.*** *Schematic illustration of selecting the best probability threshold to separate AOs and non-AOs in the validation sets.*

*X axis represents SGOs (short-gene-operons) in positive & negative validation sets; Y axis is probabilities generated by the two-state HMM. The best threshold (ThAO) was selected to maximize the separation of AOs and non-AOs.*

**5. Independent model performance evaluation**

To test the model performance, we split the 12,582 AOs in dbAO (**Table S2**) into dbAO-Train and dbAO-Test based on what Acr homologs are present in the AOs. Specifically, a total of 10,481 AOs in dbAO-Train contain homologs of 77 known Acrs published before the year 2020, and 2,101 in dbAO-Test (**Table S4**) contain homologs of 21 known Acrs published in 2021 and 2022 (**Table S5**). To study how many sequences share homology in the two data sets, we compared the protein sequences in dbAO-Test and dbAO-Train using BLASTP. We found that 24.25% (1,994 out of 8,223) of dbAO-Test proteins share >40% sequence identity and >60% coverage with dbAO-Train proteins. This suggests that the two data sets are quite different and sufficiently independent for model evaluation. It should be noted that it is expected that dbAO-Train and dbAO-Test share similar protein families (PFs), and these shared families are needed in the two-state HMM. The reason is that the key idea of the two-state HMM requires that AOs of dbAO have different protein family profiles (e.g., more AOPFs and less non-AOPFs) compared to non-AOs, and that such difference can be modeled for new AO discovery. All the previous steps (2-4) were repeated including:

1. dbAO-Train was used to build new AOPFs, non-AOPFs, dbPF, and the two-state HMM, excluding all data related to dbAO-Test.
2. Using the new two-state HMM and dbPF, the prediction probability of all AOs in dbAO-Test were calculated. AOs that passed the ThAO (-log(Prob) >3) are true positives (TP). Those with -log(Prob) <=3 are false negatives (FN). In other words, the -log(Prob) is output as a score for each AO, and only AOs with score > 3 are returned as the output.
3. The model recall/sensitivity is calculated as TP/(TP+FN), and accuracy as (TP+TN)/(TP+TN+FN+FP).

**6. Performance comparison with published tools**

We have compared the performance of AOminer against published tools: AcRanker (Eitzinger, et al., 2020), AcrFinder (Yi, et al., 2020), PaCRISPR (Wang, et al., 2020), and AcrPred (Dao, et al., 2023). For this experiment, AOminer was trained using the above constructed dbAO-Train and tested using the dbAO-Test. All the other tools were also tested using the same dbAO-Test data. Note that AOs in dbAO-Test were derived from the 21 known Acrs (**Table S5**) that are not present in the training data of AcrFinder, AcRanker, PaCRISPR, and AcrPred. Thus, using the same dbAO-Test on these tools ensures a fair comparison across all tools. AcrPred has a functional webserver, but the interface only allows submission of 3 proteins at a time, which makes conducting a large-scale performance evaluation very difficult. Unfortunately, we were not able to perform the same test on DeepAcr (Wandera, et al., 2022) and AcrNET (Li, et al., 2022). AcrNET is not a full functional tool, as only files to the trained models were provided on their Github page (<https://github.com/banma12956/AcrNET>). For DeepAcr, we attempted to install the stand-alone version from their Github site (<https://github.com/BackofenLab/DeepAcr>). However due to unresolved issues regarding environment setup, ours as well as other user’s installation so far have been unsuccessful (link to the issue: <https://github.com/BackofenLab/DeepAcr/issues/4>).

To account for the difference in the pipeline design and output/input format, each tool was run with an individualized evaluation process:

AcRanker:

AcRanker was downloaded from <https://github.com/amina01/AcRanker> and run locally. The entire viral contigs associated with the AOs of dbAO-Test were used if the contig was of viral origin. If the associated contig was of prokaryotic origin, then only the associated prophage regions were used. Annotated protein sequences by Prodigal (Hyatt, et al., 2010) of each extracted contigs/prophage regions were then provided to AcRanker as input. AcRanker ranks input protein sequences based on their potential to being Acrs. As recommended by the AcRanker paper, for each contig/prophage region, the top 5 ranked proteins were kept as Acrs. The predicted Acrs were then located in SGOs to be considered as AOs (true positives) predicted by AcRanker. The recall was calculated by dividing the number of predicted AO by the total number of AOs within dbAO-Test.

AcrFinder:

AcrFinder was downloaded from <https://github.com/HaidYi/acrfinder> and run locally. Similar to AcRanker’s evaluation approach, viral contigs or prophage regions associated with AOs of dbAO-Test were extracted and provided to AcrFinder as input. AcrFinder scans the input genomic sequence and outputs SGOs with HTH-domain containing proteins as predicted AOs. The predicted AOs were kept as true positives if they overlapped in genomic boundaries with an AO in dbAO-Test. Recall was then calculated by dividing the AcrFinder predicted AOs by the total AOs within dbAO-Test.

PaCRISPR:

PaCRISPR was run on its web server (<https://pacrispr.erc.monash.edu/>). It takes each protein sequence as input. A prediction score will be returned for the input sequence as an Acr if the score is above a preset threshold by PaCRISPR. Thus, for PaCRISPR evaluation, all proteins of dbAO-Test were submitted to PaCRISPR’s webserver as input. Based on the returned prediction scores, those that passed the default threshold were kept as Acrs. The predicted Acrs were then located in SGOs to be considered as AOs (true positives) predicted by PaCRISPR. Recall was then calculated by dividing the number of PaCRISPR predicted AOs by the total number of AOs within dbAO-Test.

AcrPred:

AcrPred has a webserver (<http://lin-group.cn/server/AcrPred/webServer.html>) that takes a protein sequence and associated PSSM as input, and outputs Prediction scores. The PSSM must be generated using psiblast with the protein as query and UniRef50 (<https://ftp.uniprot.org/pub/databases/uniprot/uniref/uniref50/>) as database (<http://lin-group.cn/server/AcrPred/pssm.html>). Due to this design, the AcrPred webserver can only predict one protein per submission, and unsuitable for large scale performance tests. Upon contacting the AcrPred team, they have agreed to run all proteins of dbAO-Test using a developer-end offline version of AcrPred. The returned Acr predictions were then located in SGOs to be considered as AOs (true positives) predicted by AcrPred. Recall was then calculated by dividing the number of AcrPred predicted AOs by the total number of AOs within dbAO-Test.

**7. Construction of dbAcr and dbHTH**

dbAcr contains the current published and experimentally characterized 89 Acr proteins. The protein sequences are stored in FASTA format.

Knowing that all known anti-CRISPR associated (Aca) proteins match the Pfam HTH HMMs, we downloaded HMMs of the Pfam HTH clan (CL0123). Only keeping HMMs with length < 150 aa (all 12 known Acas are shorter than 150 aa), and must have “HTH” or “helix turn helix” in their Pfam family descriptions (e.g., HTH_24, HTH_3). We obtained in total 89 HMMs forming the HTH database (dbHTH).

**References**

Almeida, A.*, et al.* A unified catalog of 204,938 reference genomes from the human gut microbiome. *Nature Biotechnology* 2021;39(1):105-114.

Cook, R.*, et al.* INfrastructure for a PHAge REference Database: Identification of Large-Scale Biases in the Current Collection of Cultured Phage Genomes. *PHAGE* 2021;2(4):214-223.

Dao, F.-Y.*, et al.* AcrPred: A hybrid optimization with enumerated machine learning algorithm to predict Anti-CRISPR proteins. *International Journal of Biological Macromolecules* 2023;228:706-714.

Edgar, R.C. MUSCLE: a multiple sequence alignment method with reduced time and space complexity. *BMC Bioinformatics* 2004;5(1):113.

Edgar, R.C. Search and clustering orders of magnitude faster than BLAST. *Bioinformatics* 2010;26(19):2460-2461.

Eitzinger, S.*, et al.* Machine learning predicts new anti-CRISPR proteins. *Nucleic Acids Research* 2020;48(9):4698-4708.

Finn, R.D., Clements, J. and Eddy, S.R. HMMER web server: interactive sequence similarity searching. *Nucleic Acids Research* 2011;39(suppl_2):W29-W37.

Hyatt, D.*, et al.* Prodigal: prokaryotic gene recognition and translation initiation site identification. *BMC Bioinformatics* 2010;11(1):119.

Kieft, K., Zhou, Z. and Anantharaman, K. VIBRANT: automated recovery, annotation and curation of microbial viruses, and evaluation of viral community function from genomic sequences. *Microbiome* 2020;8(1):90.

Li, Y.*, et al.* AcrNET: Predicting Anti-CRISPR with Deep Learning. *bioRxiv* 2022:2022.2004.2002.486820.

Nayfach, S.*, et al.* Metagenomic compendium of 189,680 DNA viruses from the human gut microbiome. *Nature Microbiology* 2021;6(7):960-970.

O'Leary, N.A.*, et al.* Reference sequence (RefSeq) database at NCBI: current status, taxonomic expansion, and functional annotation. *Nucleic acids research* 2016;44(D1):D733-D745.

Roux, S.*, et al.* IMG/VR v3: an integrated ecological and evolutionary framework for interrogating genomes of uncultivated viruses. *Nucleic Acids Research* 2021;49(D1):D764-D775.

Unterer, M., Khan Mirzaei, M. and Deng, L. Gut Phage Database: phage mining in the cave of wonders. *Signal Transduction and Targeted Therapy* 2021;6(1):193.

Wandera, K.G.*, et al.* Anti-CRISPR prediction using deep learning reveals an inhibitor of Cas13b nucleases. *Molecular Cell* 2022;82(14):2714-2726.e2714.

Wang, J.*, et al.* PaCRISPR: a server for predicting and visualizing anti-CRISPR proteins. *Nucleic Acids Research* 2020;48(W1):W348-W357.

Yi, H.*, et al.* AcrFinder: genome mining anti-CRISPR operons in prokaryotes and their viruses. *Nucleic Acids Research* 2020;48(W1):W358-W365.
